# Neurobiological Approach of Catatonia and Treatment Perspectives

**DOI:** 10.3389/fpsyt.2015.00182

**Published:** 2015-12-24

**Authors:** Pierre Ellul, Walid Choucha

**Affiliations:** ^1^Department of Psychiatry, Assistance Publique-Hôpitaux de Paris, Pitié-Salpétrière University Hospital, University Pierre et Marie Curie, Paris, France

**Keywords:** catatonia, emotion regulation, prefrontal cortex, GABA-A receptors, clinical neurosciences psychopharmacology

## Introduction

Catatonia was described for the first time by Kahlbaum in 1874 (1). It can be defined schematically as a motor dysregulation syndrome accompanied with a behavioral component. There are three main forms of catatonia: (i) akinetic, (ii) hyperkinetic, and (iii) malignant catatonia (2). These various phenotypes of the same syndrome led to a clinical heterogeneousness making the catatonia difficult to recognize and diagnose. It seems, however, that certain clinical signs occur with a greater frequency in catatonia. Indeed, in a study involving more than 230 catatonic patients, the “staring,” was found in more than 80% of cases. Among other frequent signs, were the immobility in 70% of cases, the mutism in 60% of patients, and the withdrawal in 50% of them (3). Various specific scales were developed to allow a more accurate diagnostic approach to catatonia. The Bush-Francis catatonia rating scale is the most commonly used one. It has many advantages: in addition to having a sensitivity of reaching 100% and a specificity between 75 and 100%, it is fast and easy to use in daily clinical practice (4). It is important to note that catatonia is a transnosographic syndrome with various underlying psychiatric and somatic causes. Most common somatic causes include epilepsy, systemic lupus erythematosus, intermittent porphyria, traumatic brain injury, dementia, encephalopathies (autoimmune, paraneoplastic, Hashimoto, etc.) (5). Furthermore, catatonia is found among 10% of psychiatric inpatients (6). Under the influence of Kraeplin, catatonia was linked for a long time exclusively with schizophrenia (7). However, recent epidemiological studies showed that schizophrenia is found only in 20% of catatonic cases while mood disorders underlie 45% of cases (8). Catatonia is also frequent in children and adolescents, particularly in autism spectrum disorders where the prevalence varies between 12 and 17% (9). Despite these clinical and epidemiological facts, few data exist concerning the exact pathophysiological mechanisms underlying this syndrome. In this paper, we will make the synthesis of the existing data concerning the neurocognitive and neurobiological mechanisms involved in the akinetic forms of catatonia.

## Prefrontal Physiology of Emotions

Moskowitz considered catatonia as an evolutionary remainder of defense strategies associated with intense fear (10). It seems that in front of predators, several survival behaviors have been developed. Among them, the most known one was the “fight or flight” strategy. In cases where none of these two options was possible, a third strategy called “tonic immobility” (TI) would be set up which consists of a tonic suspension of motor activity. This defense strategy is based on the fact that many predators are attracted by their prey’s movements. This hypothesis seems to be confirmed by the subjective experience of catatonic patients. Indeed, once remitted from their catatonia, patients report having felt invaded by a major and uncontrollable anxiety. Conversely, they do not seem to have been aware of their motor state (11). To better understand the brain abnormalities found in catatonic patients, it seems essential to focus on the neurological mechanisms involved in the physiological integration of emotions. The amygdala appears to have a central role in emotional regulation processes, particularly negative emotions, such as fear or anxiety (12). The environmental informations are conveyed to different brain areas according to each specific sensory modality (for example, visual stimuli are conveyed to occipital cortex, auditory informations to temporal cortex, etc.) and secondarily sent to the amygdala, serving as an emotional crossroad (12). To start making the link amygdala/emotions/catatonia, it may already be interesting to note that, in animals, hyperactivation of the amygdala is responsible of a freezing behavior, which is similar to TI and symptoms found in the akinetic forms of catatonia (13). Once informations are integrated in the amygdala, they may, depending on the emotional valence, activate different neural circuits. In particular, functional MRI studies found that negative emotions are associated with increased activation of the orbitofrontal cortex (OFC) and the ventromedial prefrontal cortex (VMPFC) and decreased activity of dorsolateral prefrontal cortex (DLPFC). The exact opposite activation profile occurs with positive emotions making the PFC a regulating crossroad depending on the emotion type (14). These variations in the activation/deactivation pattern seem to be modulated by the GABAergic system (15).Each of the involved brain areas is associated with specific functions. OFC is involved in decoding the emotional environmental situations and in taking decisions depending on the context (16). VMPFC is considered as a self-centered emotional integration center. It will allow, in some way, perceiving emotions (17). Regarding DLPFC role, it is implicated in cognitive processes and action planification (18). It will allow a cognitive approach in understanding emotions and a negative feedback on emotional processes, especially amygdalian ones. In other words, DLPFC performs cognitive control of emotions (19, 20). Furthermore, the DLPFC is a major integrative crossroad. Indeed, it receives informations, among others, from the posterior parietal cortex, which is itself involved in negative emotions (21). DLPFC will then project mainly on motor areas (22). It is therefore considered as a sensorimotor associative region bridging the gap between emotional cognitive perception and motor skills (23).

## Pathophysiology of Catatonia

To confirm TI hypothesis in catatonia, functional MRI studies have investigated catatonic brain activation during emotional processing. One of them compared patients who remitted from catatonia to non-catatonic psychiatric patients and finally to healthy subjects. Authors found among remitted catatonic patients, a hyperactivation of the OFC and the VMPFCs during negative emotions compared to the two other groups (24). Furthermore, statistical analysis showed a positive correlation between the hyperactivation of the OFC and behavioral/emotional symptoms and between the hyperactivation of VMPFCs and motor symptoms (24). The authors also found alterations in corticocortical connections between (i) OFC and VMPFC; (ii) between VMPFC/DLPFC and motor/premotor cortex (24). Another study examined the effect of lorazepam [a benzodiazepine known for its effectiveness in catatonia (25, 26)] on the modulation of activation patterns of the PFC during negative emotions. A decrease in OFC and VMPC hyperactivation was observed with lorazepam in successfully treated catatonic patients, leading to a regularization of the OCF activity, compared to control (27). It seems that GABA and especially the GABA-A receptors may play an important role in the pathophysiology of catatonia. One study looked at the density of GABA-A receptors as well as changes in cerebral perfusion in catatonic patients compared to non-catatonic psychiatric patients and healthy subjects. The authors found a decrease of the GABA-A receptors density in the DLPFC associated with a decrease of cerebral perfusion in prefrontal and posterior parietal cortex (28). Moreover, motor and affective symptoms were significantly associated with the decreased GABA-A receptors density in the DLPFC (28). Involvement of the DLPF in catatonia has also been demonstrated by indirect evidences, such as the therapeutic efficacy of high-frequency transcranial magnetic stimulation applied to this area (29, 30). Some authors tried to correlate brain activation changes with different catatonic symptoms, especially motor ones. A controlled study using different motor tasks (idle status, self-initiated movements, and movements on request) showed a decreased activity of the prefrontal cortex, the parietal cortex, and the supplementary motor area in catatonic patients compared to controls (31). These changes persisted even after remission. Specifically, it seems that it is the latency of late motors potentials at the frontoparietal line that is affected in catatonia with GABAergic altered sensitivity compared to control (31). These results are in agreement with the fact that catatonic patients may successfully initiate movements but present difficulties in terminating them (32, 33). Another study examined cerebral perfusion changes in catatonic patients before and after treatment with electroconvulsive therapy (ECT) and found increased perfusion in the parietal cortex after successful treatment (34). Indeed, it appears that the parietal cortex may play an important role in motricity as demonstrated by the occurrence of a catatonic state in patients with a parietal lesion (35). Considering these studies, it seems that different brain areas, in addition to the PFC, are involved in the catatonia. Neurocognitive studies showed a selective deficit in visual–spatial performances in catatonic patients compared to controls (36). These results confirmed indirectly the role of the posterior parietal cortex dysfunction in catatonia as it is broadly implicated in visual–spatial performance (37, 38). A positive correlation was also found between the activity of mirror neurons and echophenomena (echopraxia, echolalia) and their disappearance after administration of lorazepam (39). Indeed, these echo-phenomena seem to be attributed to the disinhibition of the mirror neurons, which would be related to a control deficit of the GABAergic system, within the OCF, the VMPFC, the DLPFC, and the parietal cortex (40). Moreover, glutamate seems to be involved in catatonia as well, particularly via the NMDA receptors activity. These assumptions are based primarily on the efficacy of NMDA-receptor antagonists, such as amantadine in catatonia and also in cases of catatonia related to anti-NMDA receptor encephalitis (41–44). Amantadine may act by decreasing cerebral glutamatergic activity creating a relative increase in the inhibitory GABAergic activity (45).

## Conclusion and Perspective

The exact mechanisms underlying the pathophysiology of catatonia still remain a mystery. It seems that some people are more predisposed than others to develop this syndrome. Indeed, most studies agreed on the existence of trait markers, especially GABAergic cortical dysregulation, resulting in the failure of cognitive control of emotions. When intense emotional changes generated by psychiatric disorders (depression, mania, and schizophrenia) are added to these predispositions, this would precipitate a state of TI: in other words, catatonia. Schematically, in response to negative emotions, the GABAergic inhibitory control at the OFC could not take place, leading to a deregulation in VMPFC/DLPFC balance, which would then prevent cognitive control of negative emotions by the DLPFC. In addition, the deficit in DLPFC activation would impair its associative function, and particularly its connectivity with the parietal cortex and the motor areas leading to the occurrence of the motor signs found in akinetic forms of catatonia. There are many limits to the studies mentioned above: (i) they included a small number of patients, (ii) few of them compared catatonic patients to healthy controls or to controls with psychiatric disorders, and (iii) clinical heterogeneity of catatonia was not taken in consideration in these studies. In the future, it might be interesting to develop clinico-morphological correlation studies with particular attention to the potential role of the amygdala in catatonia. This approach might open the way for new therapeutic options targeting the amygdala. For example, oxytocin seems to have a direct attenuating effect on reactions of fear and anxiety by acting directly on the amygdala (46). Other studies focusing on the role of glutamate in catatonia could pave the way for therapeutic innovations. For example, it is possible to imagine the use of drugs with dual action on both GABAergic and glutamatergic systems to treat resistant forms of catatonia. Some drugs having such properties are already available, especially acamprosate and lamotrigine which possesses this dual receptor profile (47). Rapid and accurate diagnosis and treatment of catatonia is crucial in clinical practice not only to avoid somatic complications but to avoid the development of resistance to treatment as well. Indeed, the longer catatonic symptoms last, the more will be the risk of developing resistance to treatment (48). Consequently, rapid achievement of full remission of catatonic symptoms should be an essential goal.

## Author Contributions

PE and WC participated both to research and writing of the paper in the same way.

## Conflict of Interest Statement

The authors declare that the research was conducted in the absence of any commercial or financial relationships that could be construed as a potential conflict of interest.
